# Response patterns in adventitial layer of *Echinococcus granulosus *sensu stricto cysts from naturally infected cattle and sheep

**DOI:** 10.1186/s13567-021-00936-8

**Published:** 2021-05-07

**Authors:** Christian Hidalgo, Caroll Stoore, María Soledad Baquedano, Ismael Pereira, Carmen Franco, Marcela Hernández, Rodolfo Paredes

**Affiliations:** 1 Laboratorio de Medicina Veterinaria, Escuela de Medicina Veterinaria, Facultad de Ciencias de la Vida, Universidad Andres Bello, Santiago, Chile; 2 Instituto de Ciencias Agroalimentarias, Animales y Ambientales (ICA3), Universidad de O’Higgins, San Fernando, Chile; 3 Staff Pathologist, Clinica Santa Maria, Santiago, Chile; 4 Laboratorio de Biología Periodontal y Departamento de Patología y Medicina Oral, Facultad de Odontología, Universidad de Chile, Santiago, Chile

**Keywords:** *Echinococcus granulosus*, Adventitial layer, Histopathology, Fibrosis

## Abstract

Cystic echinococcosis is a zoonotic disease caused by the metacestode of *Echinococcus granulosus *sensu lato. The disease is characterized by the development of cystic structures inside viscera of the intermediate host, mainly liver and lungs. These cysts are formed by three layers: germinal, laminated, and adventitial layer, the latter being the local host immune response. Metacestodes that develop protoscoleces, the infective stage to the definitive host, are termed fertile, whereas cysts that do not produce protoscoleces are termed non-fertile. Sheep usually harbor fertile cysts while cattle usually harbor non-fertile cysts. Adventitial layers with fibrotic resolution are associated to fertile cysts, whereas a granulomatous reaction is associated with non-fertile cysts. The aim of this study was to analyze cellular distribution in the adventitial layer of fertile and non-fertile *E. granulosus *sensu stricto cysts found in liver and lungs of cattle and sheep. A total of 418 cysts were analyzed, 203 from cattle (8 fertile and 195 non-fertile) and 215 from sheep (64 fertile and 151 non-fertile). Fertile cysts from cattle showed mixed patterns of response, with fibrotic resolution and presence of granulomatous response in direct contact with the laminated layer, while sheep fertile cysts always displayed fibrotic resolution next to the laminated layer. Cattle non-fertile cysts display a granulomatous reaction in direct contact with the laminated layer, whereas sheep non-fertile cysts display a granulomatous reaction, but in direct contact with the fibrotic resolution. This shows that cattle and sheep cystic echinococcosis cysts have distinct local immune response patterns, which are associated to metacestode fertility.

## Introduction

Cystic Echinococcosis (CE) is a zoonotic parasitical infection caused by the metacestode of *Echinococcus granulosus *sensu lato. CE has worldwide distribution, with higher prevalence in human and animal hosts in south Europe, middle Asia, north and south Africa and south America [1, 2]. It is recognized by the World Health Organization as a Neglected Tropical Disease (NTDx) [3]. *Echinococcus granulosus *sensu lato has an indirect life cycle, with herbivores as intermediate hosts and canids as definitive hosts. Humans are considered as dead-end hosts. In the domestic life cycle, ruminants, such as sheep and cattle, are the primary intermediate hosts, and dogs are the definitive hosts. Canids release eggs with infective oncospheres through their feces. Intermediate and dead-end hosts ingest the oncospheres which then migrate mainly to the host’s lungs and liver, where they establish a CE cyst, formerly known as a hydatid cyst [4]. CE cysts are composed by an inner germinal layer (GL) and an outer laminated layer (LL), that are of parasite origin, and an adventitial layer (AL) that is made by the host immune response [5]. The germinal layer is a cellular layer that forms the infective structure called protoscolex (PSC), which are contained in the cyst fluid; the laminated layer is a modified extracellular matrix synthesized by the germinal layer [6]. The adventitial layer is composed by immune cells from innate and adaptive responses followed by tissue from the organ where the cyst is located [7]. Some CE cysts do not have PSC and thus are considered non-fertile, as they are not able to continue with the parasite’s life cycle [2]. CE cyst fertility percentage varies greatly within studies and different species of hosts. Even within the same species, like cattle, fertility ranges from 0 to 96% of CE cysts [8]. Factors such as host species, breed and age, cyst location and size, or genotype of the parasite in some degree are associated with fertility [9]. However, the cause of fertility or non-fertility in CE cysts it is not yet fully understood. Current evidence aims to factors beyond host genetics and parasite localization for the survival and development of *E. granulosus *sensu lato within its mammal host. It is necessary to consider the local immune responses to individual cysts in addition to the overall systemic responses by individual hosts [10]. This is further supported by the fact that CE cyst fertility and adventitial layer composition appears to be independent from *E. granulosus *sensu lato genotype and *E. granulosus *sensu stricto haplotype on cattle and sheep, while the cellular composition differs depending on the fertility of CE cysts: non-fertile CE cysts are characterized by an inflammatory response and fertile CE cysts predominantly have a fibrous reaction [5, 11]. The adventitial layer has been described in different intermediate hosts and commonly it can be composed by infiltrated immune cells and fibrotic tissue, and its function was originally described as a separation between the organ tissue and the parasitic cyst [12]. This layer is characterized by the infiltration of T and B cells, macrophages and the presence of immunoglobulins [5, 7, 13, 14]. Immunoglobulin G has been studied on GL of fertile and non-fertile cattle CE cysts, where IgG1 was predominantly present on non-fertile CE cysts, while IgG1 and IgG2 were present on fertile and non-fertile CE cysts [13, 14]. In the case of adaptive immune cell response, there is infiltration of T and B lymphocytes, but with a predominance of T lymphocyte population both in sheep CE cysts and in cattle CE cysts, following the same pattern of cell distribution in both animal [7, 15]. Macrophages that infiltrate the adventitial layer form a palisade surrounding the CE cysts in cattle, while in sheep it is less frequent. In non-fertile CE cysts, macrophages are located surrounding the laminated layer, unlike fertile CE cysts, where the macrophages are predominantly near the fibrosis reaction [5, 16]. As cattle CE cysts usually are non-fertile and sheep CE cysts are commonly fertile [17–19], we focused our work on the cellular distribution and extracellular matrix changes in adventitial layer of fertile and non-fertile CE cysts found in liver and lungs of cattle and sheep.

## Materials and methods

This study was approved by the Universidad Andres Bello bioethics committee, protocol number 012/2019.

### Samples

Cattle and sheep CE cysts were obtained from abattoirs in different locations. After official veterinary inspection, the gold standard for CE diagnosis in intermediate hosts [20], CE cysts were excised from liver and lungs and placed in independent labeled plastic bags, which were stored in an isothermal cooler. After the slaughter, samples were transported to the laboratory for processing. Table 1 shows sample distribution according to intermediate host species and anatomical localization.

**Table 1:** **CE cyst fertility according to anatomical location in cattle and sheep**

	Lung	Liver
Cattle		
Fertile CE cyst	7	1
Non-fertile CE cyst	110	85
Sheep		
Fertile CE cyst	34	30
Non-fertile CE cyst	95	56

Data are expressed as absolute frequencies. *P* value for cattle = 0.1418; *P* value for sheep = 0.2232

### Sample processing and fertility evaluation

Each CE cyst was measured with a caliper, the cyst fluid was aseptically aspirated and stored, and using a disposable scalpel, the cyst was opened. To evaluate CE cyst fertility, protoscoleces (PSC) or samples of the germinal layer were observed under a light microscope with trypan blue exclusion test. Only CE cysts with viable PSC were considered as fertile; all other CE cysts were considered non-fertile.

### Genotyping

Mitochondrial DNA from germinal layer samples was obtained with WIZARD Plus SV Genomic Purification Systems kit (PROMEGA). Genotyping was performed by amplifying and sequencing the full length of the cytochrome C oxidase subunit (cox1) gene, as previously described [11]. Sequences were aligned with the Eg01 haplotype (Accession No. JQ250806) [21], and only *E. granulosus *sensu stricto samples were included in the study.

### Histological processing

For each CE cyst, two samples of cyst wall (comprising germinal, laminated and adventitial layer as well as organ tissue) were cut and placed in histological cassettes. Samples were dehydrated in an automatic tissue processor with an increasing alcohol gradient, cleared with NeoClear® (Merck) and embedded in paraffin. While crafting the paraffin blocks, tissue orientation was made in order to obtain cuts with the germinal, laminated and adventitial layers in succession. Blocks were cut in 5 µm thick sections and stained with haematoxylin–eosin (HE) for an initial screening. Samples with the three layers in succession were considered suitable for histochemical analysis.

### Van Gieson staining

To evaluate fibrosis in tissue sections, the Van Gieson (VG) histochemical technique was performed using the DiaPath (Catalog No. 010237) kit following the manufacturer’s instructions.

### Histopathological analysis

Both HE and VG slides were analyzed blindly by the authors and trained pathologist to evaluate inflammatory responses and their localization regarding the fibrotic response in the adventitial layer, using the laminated layer as a reference point.

### Statistical analysis

To evaluate if a response pattern was associated with host species, CE cyst fertility or anatomical site, Fisher exact test was performed, with the Freeman-Halton extension, combining response patterns II and III in cattle and response patterns I and IV in sheep. Associations were considered statistically significant if *P* < 0.05.

## Results

There was no statistically significant association between CE cyst fertility and anatomical location in cattle (*P* = 0.1418) and sheep (*P* = 0.2232) (Table 1).

From 418 CE cyst samples sequenced, after histological processing, samples were included when the 3 tissue layers (germinal, laminated, and adventitial) were obtained in succession. The total number of samples included for morphohistological analysis were 185 cattle CE cysts (6 fertile and 179 non-fertile) and 199 sheep CE cysts (82 fertile and 117 non-fertile).

**Figure 1:**
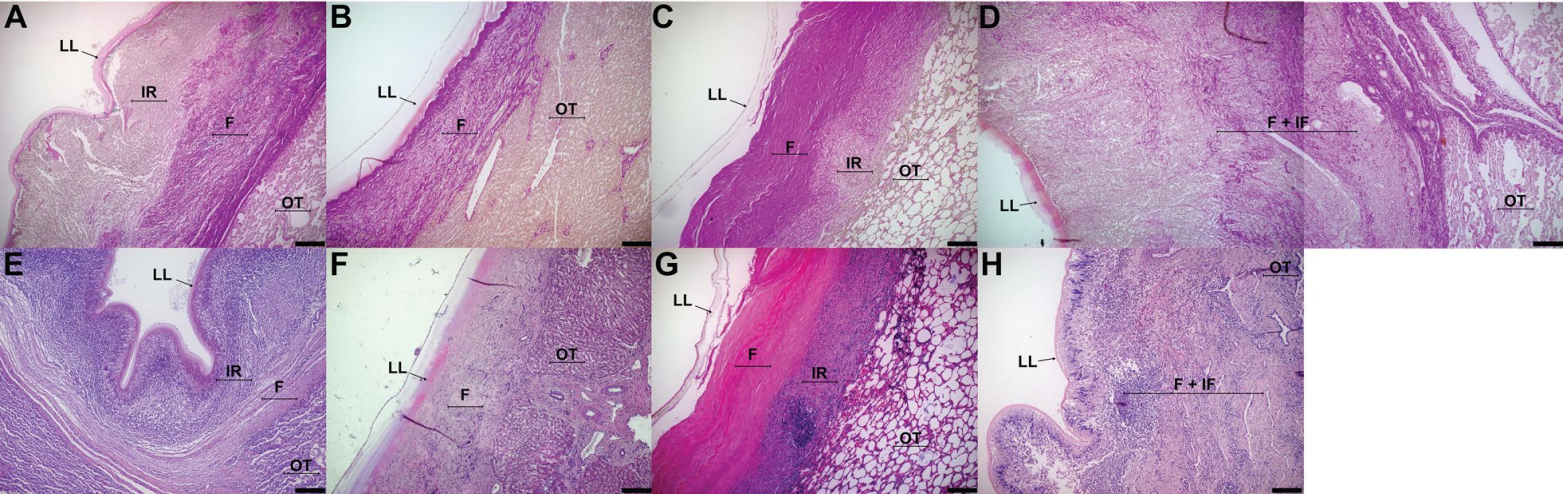
**Response patterns of adventitial layer of CE cysts**. **A**, **D** Pattern I, fibrosis is found between inflammatory reaction and organ tissue. **B**, **F** Pattern II, fibrosis is found between the laminated layer and organ tissue, and no obvious inflammatory reaction is present. **C**, **G** Pattern III, fibrosis is found between the laminated layer and inflammatory reaction. **D**, **H** Pattern IV, there is diffused fibrosis within the inflammatory reaction. LL = Laminated layer, IR = inflammatory reaction, OT = organ tissue, F = fibrosis. A, B, C, D = Van Gieson stain. E, F, G, H = Haematoxylin–eosin stain. Size bar: 200 µm. CE = Cystic echinococcosis.

Histological analysis of CE cyst tissue sections revealed four distinct response patterns to be present in cattle and sheep slides. Pattern I occurs when the fibrotic capsule is found between the inflammatory response and the organ tissue. Pattern II occurs when fibrosis is found between the laminated layer and organ tissue, and no obvious inflammatory reaction is present. Pattern III occurs when the fibrotic capsule is found between the laminated layer and the inflammatory response. Finally, Pattern IV occurs when the fibrotic capsule is intertwined with the inflammatory response. A representative image of each pattern is shown in Figure 1.

**Figure 2:**
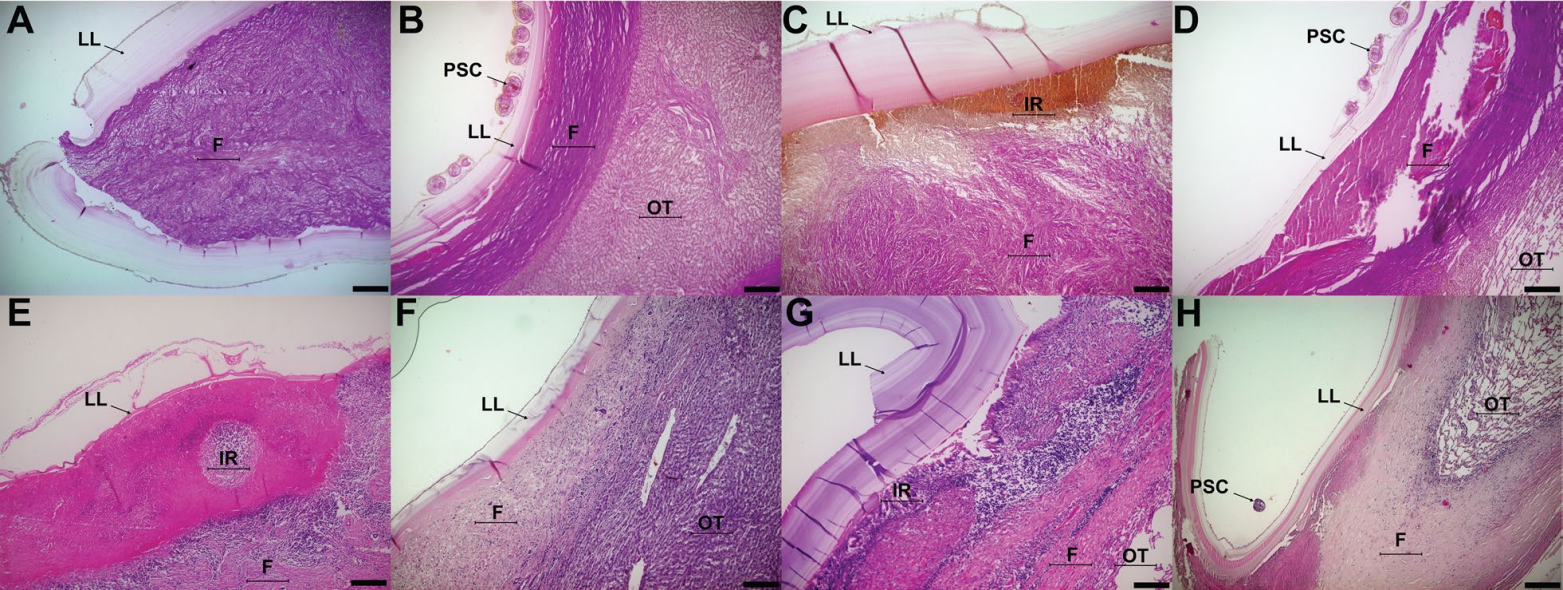
**Response patterns between cattle and sheep fertile CE cysts**. **A**, **E** Cattle fertile liver CE cyst (Pattern III), **B**, **F** Sheep fertile liver CE cyst (Pattern II), **C**, **G** Cattle fertile lung CE cyst (Pattern I), **D**, **H** Sheep fertile lung CE cyst (Pattern II). LL = Laminated layer, IR = inflammatory reaction, OT = organ tissue, F = fibrosis. A, B, C, D = Van Gieson stain. E, F, G, H = Haematoxylin–eosin stain. Size bar: 200 µm. CE = Cystic echinococcosis.

When analyzing these response patterns in fertile cattle CE cysts, liver CE cysts displayed patterns II and III, whereas lung CE cysts only displayed pattern I. In sheep, both liver and lung fertile CE cysts displayed patterns II and III (but never pattern I). Response pattern IV was not found in fertile CE cysts. A representative image of each kind of sample is shown in Figure 2.

**Figure 3:**
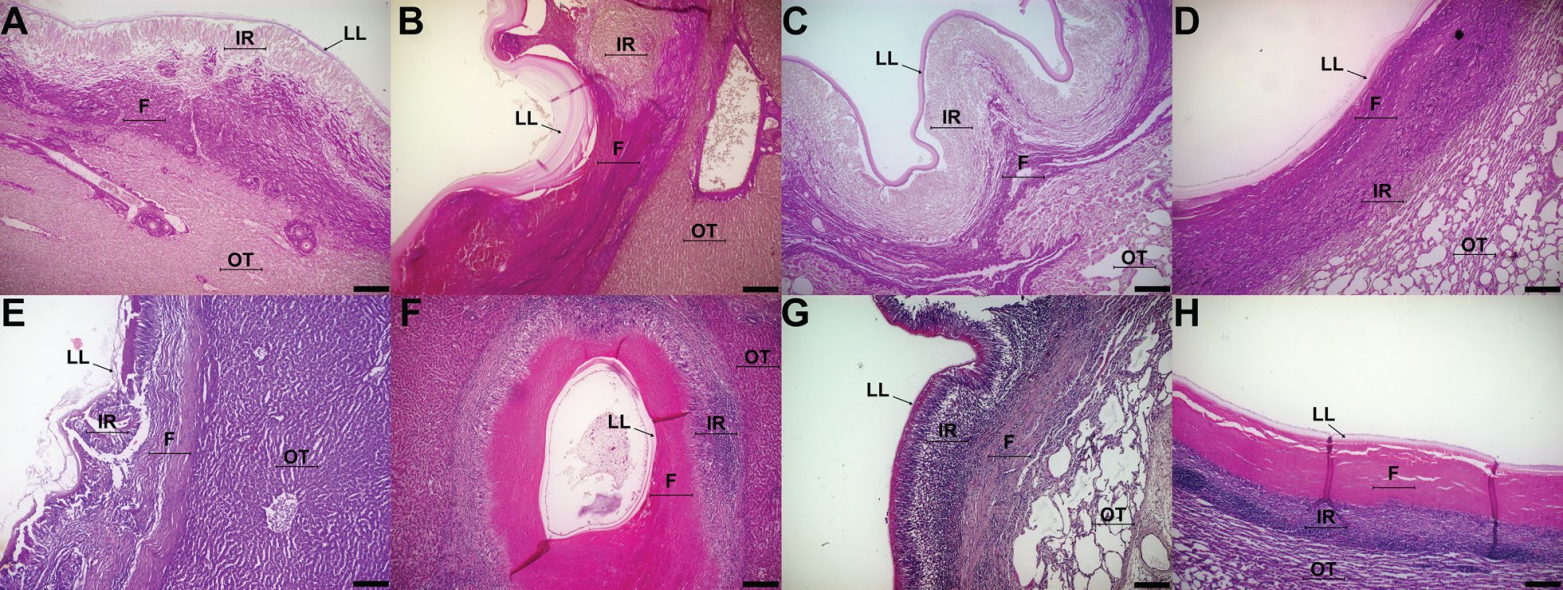
**Fibrosis patterns between cattle and sheep non-fertile CE cysts**. **A**, **E** Cattle non-fertile liver CE cyst (Pattern I), **B**, **F** Sheep non-fertile liver CE cyst (Pattern III), **C**, **G** Cattle non-fertile lung CE cyst (Pattern I), **D**, **H** Sheep non-fertile lung CE cyst (Pattern III). LL = Laminated layer, IR = inflammatory reaction, OT = organ tissue, F = fibrosis. A, B, C, D = Van Gieson stain. E, F, G, H = Haematoxylin–eosin stain. Size bar: 200 µm. CE = Cystic echinococcosis.

Regarding non-fertile cysts, cattle lung and liver CE cysts display predominantly a response pattern I, with the additional occurrence of pattern IV. Non-fertile sheep cysts show patterns II and III predominantly. A representative image of each kind of sample is shown in Figure 3.

Statistical analysis shows that cattle non-fertile CE cysts, have a response pattern I whereas sheep CE cysts show pattern II predominantly in fertile CE cysts while pattern III prevails in non-fertile CE cysts (Table 2). Regarding anatomical location and response pattern, there was no statistically significant association between response pattern and affected organ in cattle (*P* = 0.0731) and sheep (*P* = 1) (Table 3).

**Table 2:** **CE cyst fertility according to the response patterns within adventitial layer in cattle and sheep**

		Pattern I	Pattern II		Pattern III		Pattern IV
Cattle							
Fertile		3	2		1		0
Non-Fertile		168	1		0		10
Sheep							
Fertile		0	61		21		0
Non-Fertile		1	23		92		1

The numbers represent how many CE cysts featured each response pattern

Data are expressed as absolute frequencies. *P* value for cattle = 0.00014; *P* value for sheep < 0.0001

**Table 3:** **Response patterns within the adventitial layer of lung and liver CE cysts**

		Pattern I		Pattern II		Pattern III		Pattern IV
Cattle								
Lung		99		3		0		9
Liver		72		0		1		1
Sheep								
Lung		1		52		71		0
Liver		0		32		42		1

Data are expressed as absolute frequencies. *P* value for cattle = 0.0731; *P* value for sheep = 1

## Discussion

For many years, the adventitial layer of CE cysts has been described as a host-derived fibrous capsule surrounding the parasite [22], and while this remains true for many fertile CE cysts, currently the adventitial layer is considered to be the result of the local immune response against fertile and non-fertile CE cysts, with granulation tissue, plasma cells, lymphocytes, eosinophils, and other innate immune cells [5, 16, 23]. Previously it was reported that cattle CE cyst fertility was associated with the local inflammatory response, with fertile CE cysts with low PSC viability showing higher levels of granulomatous reaction near the laminated layer than high PSC viability cysts [5]. We now report that sheep non-fertile CE cysts display granulomatous reaction, although not directly in contact with the laminated layer as in their cattle counterparts. This reaction was previously reported by Barnes et al. [16], where it was found that sheep CE cysts display a granulation tissue surrounding the fibrotic reaction, although they did not associate the presence of this kind of reaction with CE cyst fertility. Liver and lung CE cysts found in humans show a response pattern III in the adventitial layer, however, association of inflammatory response with CE cyst fertility was not evaluated [24].

Either in cattle or sheep CE cysts, the presence of a granulomatous reaction is indicative of a foreign body response, which has been widely studied in the biomaterials field [25–27], where after an acute inflammatory reaction, there is chronic inflammation, followed by either granulomatous tissue or the development of a fibrous capsule [27]. Here we report that in non-fertile CE cysts, this fibrous capsule changes its location; in cattle it is found surrounding the chronic inflammation tissue, whereas in sheep is found directly surrounding the laminated layer.

The laminated layer is the main parasite tissue in contact with the local immune response, and it has been shown to elicit mostly anti-inflammatory responses [28–32], which is coherent with results from sheep CE cysts, but it is not consistent with what can be found in cattle CE cysts. One possible explanation could be that macrophage differentiation differs between M1 or M2 phenotypes in cattle and sheep, respectively, since M1 macrophages have been associated with granulomatous tissue [33] and M2 macrophages with fibrous tissue response [34]. The predominance of either macrophage population in adventitial layer of cattle and sheep CE cysts should be further investigated.

In cattle, macroscopically, fertile CE cysts have a white and thick laminated layer that easily detaches from the adventitial layer, whereas non-fertile CE cysts have a yellow and thin laminated layer firmly attached to the adventitial layer [35]. This difference could be attributed to a higher concentration of host proteins in the germinal layer, such as immunoglobulins [14], as well as the chronic inflammation which is in direct contact with the laminated layer in cattle CE cysts [5]. The presence of fibrosis in direct contact with the laminated layer also explains why it easily detaches from the adventitial layer in sheep CE cysts and fertile cattle CE cysts. The international consensus for *Echinococcus* nomenclature defined that non-fertile CE cysts are metacestodes that do not contain viable protoscoleces [2], our results support this definition, as response pattern III (inflammation surrounding the fibrous capsule) is present predominantly in non-fertile sheep CE cysts, further associating the host immune response with CE cyst fertility. This association is remarkable and highlights the detrimental effect that granulomatous responses have on *E. granulosus *sensu stricto metacestodes.

Since the fibrotic capsule further isolates the metacestode from the inflammatory response, fibrolytic therapies could be developed for the medical management of the disease, especially in cases where surgery is not an option. It has been shown that adventitial layer disorganization with invasion of inflammatory cells is associated with parasite death following oxfendazole treatment [22], and CD248 has been shown to be a molecule critical for fibrosis development in mice [36], making plausible a therapy targeting macrophages or fibroblasts.

In summary, we show that fertile and non-fertile CE cysts from cattle and sheep show a distinct response pattern. Fertile cattle CE cysts have a fibrous capsule surrounding the laminated layer, followed by either the normal organ tissue or an inflammatory reaction. Non-fertile cattle CE cysts have an inflammatory reaction surrounding the laminated layer, followed by a fibrous capsule. Fertile and non-fertile sheep CE cysts have a fibrous capsule surrounding the laminated layer, followed by normal organ tissue or inflammatory reaction, respectively. This remarkable difference shows the complex host–pathogen interaction between the metacestode and its mammalian host, expanding the available knowledge regarding CE cyst fertility.

## Data Availability

The datasets during and/or analyzed during the current study are available from the corresponding authors on reasonable request.

## Abbreviations

CE: Cystic echinococcosis
GL: Germinal layer
LL: Laminated layer
AL: Adventitial layer
PSC: Protoscolex
HE: Haematoxylin–eosin
VG: Van Gieson
